# Rumpel-Leede Phenomenon Secondary to Acute Epstein-Barr Virus Infection in a Young Adult

**DOI:** 10.7759/cureus.97986

**Published:** 2025-11-27

**Authors:** Miguel Cervantes, Alexandre Cervantes, Disha Hayagreev, Pawel Chomicki

**Affiliations:** 1 Acute Internal Medicine, Hampshire Hospitals NHS Foundation Trust, Basingstoke, GBR; 2 Emergency Medicine, Hampshire Hospitals NHS Foundation Trust, Basingstoke, GBR; 3 Cardiology, Portsmouth Hospitals University NHS Trust, Portsmouth, GBR

**Keywords:** capillary fragility, epstein-barr virus, glandular fever, infectious mononucleosis, maculopapular rash, petechiae, petechial rash, rumpel-leede phenomenon, rumpel-leede sign

## Abstract

Rumpel‑Leede sign (RLS) or phenomenon describes the sudden appearance of well-demarcated petechiae distal to where a tourniquet or sphygmomanometer cuff have been applied. This can be a result of dermal capillary rupture in the context of increased venous hydrostatic pressure due to external compressive forces and capillary fragility or an underlying coagulopathy or thrombocytopaenia.

We report the case of a 20‑year‑old male patient who initially presented with a pruritic widespread maculopapular rash following a course of Penicillin V in the context of an undiagnosed Epstein-Barr virus (EBV) infection and subsequently developed a petechial rash consistent with RLS, following application of a tourniquet prior to venepuncture. Laboratory investigations showed a normal platelet count and coagulation profile. EBV serology was consistent with acute EBV infection. No specific treatment was indicated or provided for the Rumpel-Leede phenomenon, which gradually resolved. This case highlights that RLS may occur in the setting of acute EBV infection even in the absence of thrombocytopenia or coagulopathy and underscores the importance of recognition to avoid unnecessary alarm or investigations.

## Introduction

The Rumpel‑Leede sign (RLS) is a rare phenomenon that represents the appearance of a well-demarcated petechial rash distal to an area where pressure has been applied, often by a tourniquet or sphygmomanometer cuff [1]. Epstein-Barr virus (EBV) is a common cause of infectious mononucleosis, typically presenting with fever, pharyngitis, lymphadenopathy, and occasionally hepatosplenomegaly [2]. Cutaneous manifestations are common, particularly when β‑lactam antibiotics are administered during acute infection: a pruritic maculopapular rash is well described. Although the classic “amoxicillin‑rash” in EBV is a well-documented phenomenon, the occurrence of RLS in acute EBV infection, as far as we are aware, has only been documented in one other case report but in the context of an associated thrombocytopaenia [3]. We present a case in a young, healthy adult with infectious mononucleosis, also known as glandular fever, and RLS with normal platelet count and coagulation profile, to highlight that this phenomenon can also occur in acute EBV infection without thrombocytopaenia and further investigations to exclude an underlying bleeding diathesis may therefore be avoided.

## Case presentation

A 20‑year‑old male patient with a background of seasonal allergic rhinitis and mild asthma on no regular medications presented to the emergency department with a five‑day history of a widespread pruritic maculopapular rash. A week earlier he had developed a sore throat and was prescribed penicillin V by his general practitioner (GP) for presumed tonsilitis. The maculopapular rash appeared after four days of taking penicillin. The GP switched the patient from penicillin V to clarithromycin, started fexofenadine, and prescribed a five‑day course of prednisolone 30 mg once daily. Despite this, the pruritic rash persisted, and the patient presented to the emergency department. On examination, there was a blanching maculopapular rash predominantly affecting the patient’s torso, limbs and face; the palms and soles were spared and mucous membranes unaffected. The patient was afebrile and haemodynamically stable (respiratory rate 19 breaths per minute, SpO₂ saturation 98% on room air, blood pressure (BP) 152/88 mmHg, heart rate 97 beats per minute). The patient’s main complaint was pruritus associated with the rash. Tonsils were mildly erythematous without exudate; there was no meningism, no lymphadenopathy, and the remainder of the clinical examination was unremarkable. 

**Figure 1:**
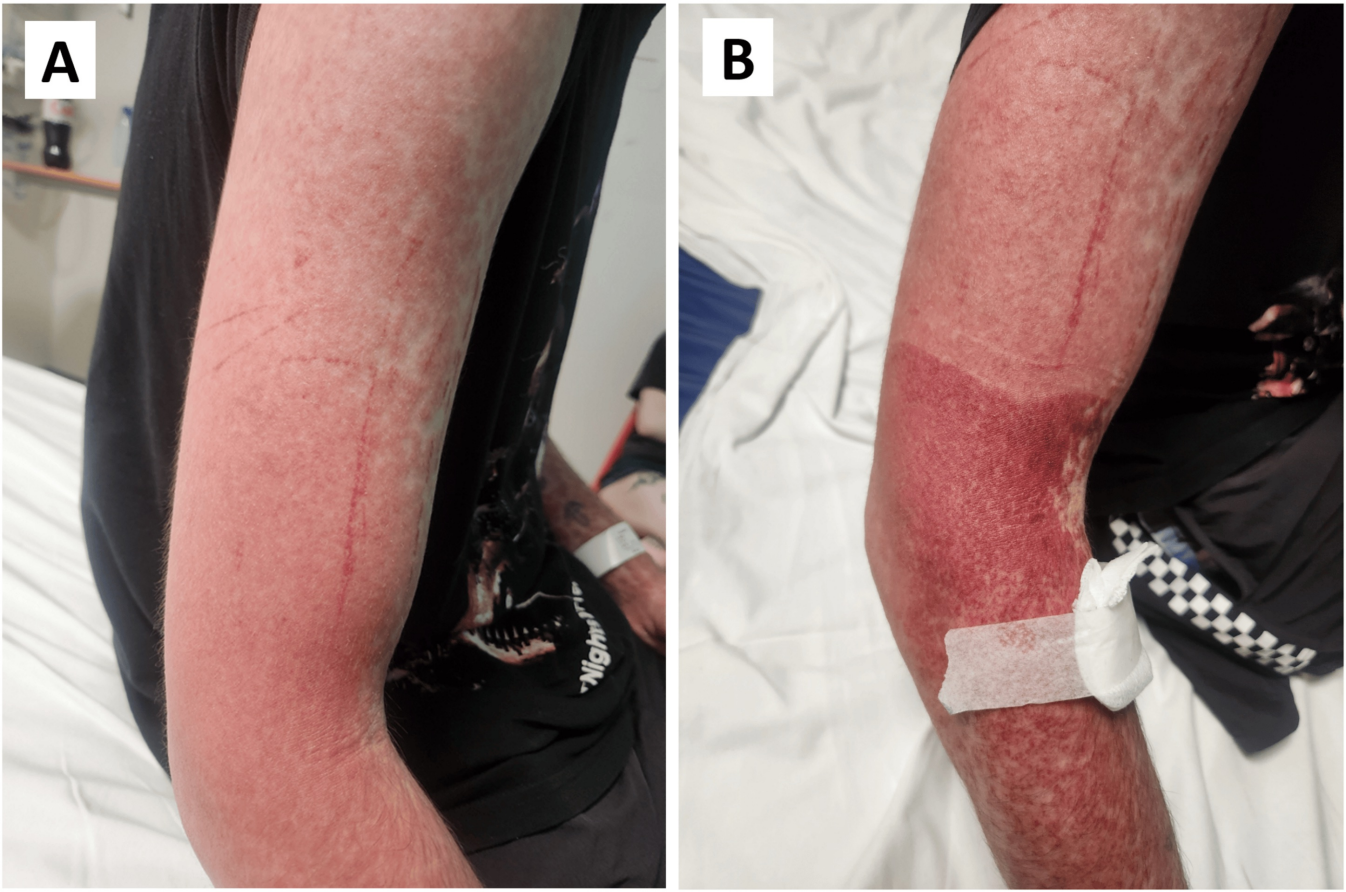
Patient’s right upper limb before (A) and after (B) application of tourniquet for venepuncture Picture on the left (A) depicts the maculopapular rash the patient initially presented with, while the image on the right (B) represents RLS, a petechial purpuric rash with a well delineated margin immediately distal to the site of tourniquet application. RLS: Rumpel-Leede sign

The patient was needle-phobic. Prior to venepuncture, a tourniquet was applied to the right upper arm. After blood collection and upon release of the tourniquet, a sharply demarcated non-blanching petechial rash appeared distal to the where the tourniquet had been applied and extending down the arm, as pictured in Figure 1. This was identified as the Rumpel‑Leede phenomenon. 

Laboratory tests revealed a marginally raised C-reactive protein (CRP) of 9 mg/L, normal eosinophil count of 0.00 ×10⁹/L, lymphopaenia of 1.07 ×10⁹/L, and normal platelet count of 384 ×10⁹/L. Coagulation profile was in normal range with a prothrombin time (PT) of 13.2 seconds and activated partial thromboplastin time (aPTT) of 34.1 seconds. A mildly raised alanine aminotransferase (ALT) at 69 U/L was noted and the remaining liver function tests were in normal range. Renal function and electrolytes were in the normal range. Table 1 below lists further investigation results and corresponding reference ranges.

**Table 1:** Laboratory investigation results. The table lists relevant full blood count and biochemistry laboratory investigations with corresponding reference ranges. CRP: C-reactive protein, PT: Prothrombin time; aPTT: Activated partial thromboplastin time; ALT: Alanine aminotransferase; ALP: Alkaline phosphatase; eGFR: Estimated glomerular filtration rate

Investigation	Results	Reference range
CRP	9 mg/L	0-5 mg/L
Eosinophils	0.00 x10^9^/L	0-0.8 x10^9^/L
Lymphocytes	1.07 x10^9^/L	0-0.3 x10^9^/L
Platelets	384 x10^9^/L	150-500 x10^9^/L
White Cell Count	6.3 x10^9^/L	4-11 x10^9^/L
Haemoglobin	155 g/L	130-180 g/L
PT	13.4 seconds	12.4-14.6 seconds
aPTT	34.1 seconds	26.3-37.7 seconds
Fibrinogen	3.7 g/L	1.89-4.3 g/L
ALT	69 U/L	0-50 U/L
ALP	93 U/L	30-130 U/L
Bilirubin	11 umol/L	0-21 umol/L
eGFR	>90 mls/minute	60-150 mls/minute
Creatinine	50 umol/L	59-104 umol/L
Potassium	4.1 mmol/L	3.5-5.3 mmol/L
Sodium	140 mmol/L	133-146 mmol/L
Urea	3.5 mmol/L	2.5-7.8 mmol/L

Additionally, a throat swab sent for culture grew only commensals and no β‑haemolytic Streptococci were isolated. Blood cultures were negative. Serology for EBV in the form of viral capsid antigen immunoglobulin M (IgM) and immunoglobulin G (IgG) was positive, supporting a diagnosis of acute EBV infection. The mildly elevated ALT was secondary to EBV infection. The patient was managed with antihistamines for symptomatic relief of the pruritus associated with the initial antibiotic-related maculopapular rash. No specific treatment was indicated or given for the Rumpel-Leede phenomenon. No further antibiotics were initiated. On outpatient follow‑up a week later, the rash continued to gradually resolve; the patient declined repeat blood tests due to needle‑phobia and was discharged back to the continued care of his GP to repeat liver function tests in four weeks to ensure resolution.

## Discussion

The initial rash was most likely antibiotic-induced in the context of acute EBV infection, a well-known complication which is classically associated with antibiotics, especially β-lactams, given during acute infection [4-7]. In our case, the timeline of penicillin V use followed by rash onset is consistent with this phenomenon and accounts for the initial maculopapular rash the patient presented with.

RLS occurs when external circumferential compression, usually exerted by a tourniquet or blood-pressure cuff, causes increased capillary hydrostatic pressure distal to the occlusion site and leads to the appearance of petechiae due to dermal capillary rupture, in the setting of capillary fragility. Predisposing states for capillary fragility include thrombocytopenia, platelet dysfunction, hypertension, diabetes mellitus, renal disease, corticosteroid therapy, and acute infections [8].

In this case, the absence of thrombocytopenia or other obvious comorbidities suggest capillary fragility due to acute infection may have been the most likely precipitant. RLS has also been associated with other viral infections such as COVID-19 and parvovirus B19 [9,10].

This case is noteworthy for the occurrence of Rumpel‑Leede phenomenon in the context of acute EBV infection, with normal platelet count and coagulation parameters. There has been a prior documented case of EBV-associated Rumpel-Leede phenomenon but in the context of thrombocytopaenia, which was absent here [3].

EBV-induced thrombocytopaenia is relatively common and usually mild, but can be severe in rarer cases [11,12].

We speculate that another possible contributing mechanism could potentially be platelet-dysfunction despite normal counts, due to the underlying viral infection, although no platelet function tests were carried out in our case to evaluate this [13].

## Conclusions

We report a case of RLS in a previously healthy 20‑year‑old male patient with acute EBV infection, occurring despite normal platelet and coagulation parameters. The phenomenon appears benign and self‑resolving, and in the right clinical context, EBV should be considered as a possible cause. This awareness may reduce unnecessary investigations looking for possible underlying haematological disorders or vasculitis as the cause and reassure clinicians and patients that no specific treatment or follow-up is required.
